# Rapid progression from complete molar pregnancy to post-molar gestational trophoblastic neoplasia: a rare case report and literature review

**DOI:** 10.3389/fonc.2023.1303249

**Published:** 2023-12-15

**Authors:** Jing Qian, Kaoma Gracious, Liping Sun

**Affiliations:** ^1^ Department of Gynecology, Affiliated Hangzhou First People’s Hospital, Westlake University School of Medicine, Hangzhou, Zhejiang, China; ^2^ International Education College, Zhejiang Chinese Medical University, Hangzhou, Zhejiang, China

**Keywords:** gestational trophoblastic disease, gestational trophoblastic tumor, hydatidiform mole, complete hydatidiform mole, ultrasound

## Abstract

**Background:**

Post-molar gestational trophoblastic neoplasia (pGTN) develops in about 15% to 20% of complete hydatidiform mole (CMH). Commonly, pGTN is diagnosed based on hCG monitoring following the molar evacuation. To date, no detailed information is available on how fast can pGTN develop from CHM. However, the concurrence of CHM and pGTN is extremely rare.

**Case presentation:**

A 29-year-old woman presented to the gynecology department with irregular vaginal bleeding and an elevated hCG serum level. Both ultrasound and MRI showed heterogeneous mass in uterine cavity and myometrium. Suction evacuation was performed and histologic examination of the evacuated specimen confirmed complete hydatidiform mole. Repeated ultrasound showed significant enlargement of the myometrium mass one week after the evacuation. pGTN with prognostic score of 4 was then diagnosed and multi-agent chemotherapy regimen implemented with a good prognosis.

**Conclusion:**

In rare cases, CMH can rapidly progress into pGTN. Imaging in combination with hCG surveillance seems to play a vital role guiding timely diagnosis and treatment in the specific condition. Low-risk gestational trophoblastic neoplasia (GTN) should be managed stratified according to the individual situation.

## Introduction

Post-molar gestational trophoblastic neoplasia, which is referred to as pGTN, is defined as the malignant change of hydatidiform mole, including invasive mole and choriocarcinoma (1). pGTN is signified by a plateaued or rising serum hCG concentration post-evacuation (2). Hydatidiform mole can be classified ​as complete or partial based on differences in morphology, karyotype, and malignant potential. Studies showed that the incidence of developing pGTN is approximately 15~20% from complete hydatidiform moles (CHMs) and less than 1~5% from partial hydatidiform moles (1, 3). Generally, a diagnosis of pGTN is made by hCG monitoring post-evacuation of the uterine molar tissue. The FIGO criteria for diagnosis of pGTN including: the plateau of hCG lasts for four measurements over a period of 3 weeks or longer; a rise in hCG for three consecutive weekly measurements over a period of 2 weeks or longer; histological evidence of choriocarcinoma (4). Although outcomes for most GTN arising from molar pregnancies are excellent, a few women die from the disease, mainly because of late diagnosis or drug resistance. Timely diagnosis and management of this disorder is of great importance, which contributes to the application of chemotherapy in-time and the reduction of severe complications and deaths.

To date, there is no detailed information regarding how fast malignant transformation occurs after molar pregnancy. Following a molar evacuation, studies reported that either an invasive mole develops after an average of 6 months in contrast to the development of a choriocarcinoma after an average of 13 months (4, 5). The risk of developing any pGTN from CHM within 1 month is low, only isolated cases had been reported (6–8). Here, we report an extremely rare case of a simultaneous presentation of CMH and pGTN, which may be misdiagnosed with choriocarcinoma arising from previous term delivery. Imaging played a vital role in the diagnosis of the case instead of usual hCG surveillance after molar tissue evacuation. Detailed imaging of the mass was provided, specifically the pelvic ultrasound images accompanying by uterine arteriovenous fistula. The patient was successfully cured by multi-agent combination chemotherapy regime and uterine arteriovenous fistula gradually disappeared during the course. This case makes us aware of the heterogeneous presentations of pGTN and highlights the following points: 1. CHM and pGTN can concurrently occur. In this rare condition, conventional hCG monitoring criteria fails to detect the pGTN timely. Imaging plays a crucial role in identifying and guiding the management of this rare condition. 2. GTN should be treated according to the individual situation, a part of low-risk GTN may need to be administered with multi-agent chemotherapy. 3. Uterine arteriovenous fistula can occur simultaneously with GTN, which may indicate an underlying association between the two disorders.

## Case presentation

**Figure 1:**
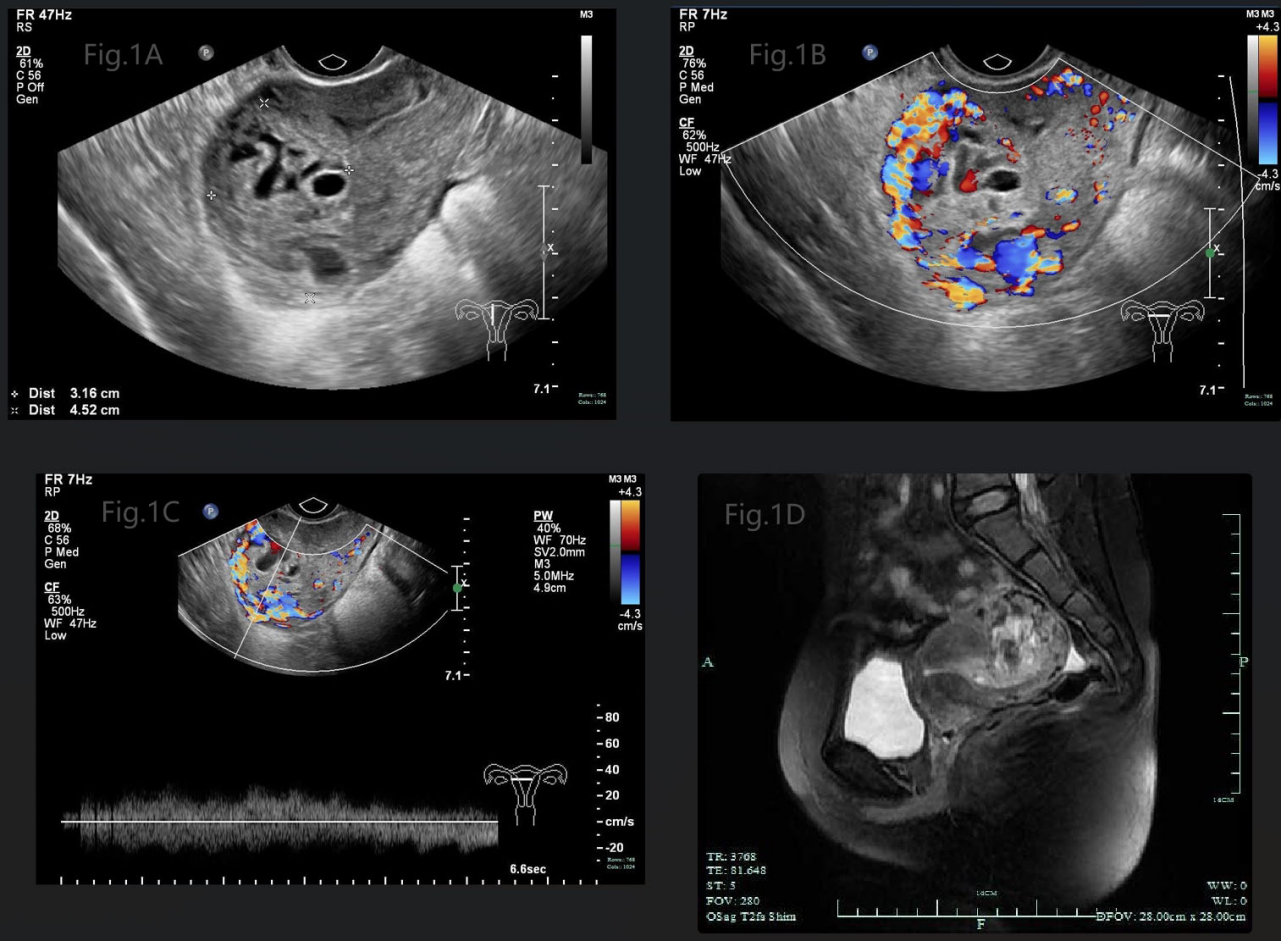
**(A)** Gray-scale pelvic sonography displayed a mixed echogenic mass in the uterine cavity, partially invading the myometrium, with honeycomb like internal echoes. **(B)** Color Doppler showed that the mass was filled with abundant blood flow signals. **(C)** Spectrum of arteriovenous fistula was measured in pulsed-wave Doppler. **(D)** Pelvic MRI scan depicted heterogeneous mass in uterine cavity with hemorrhage, invading to the myometrium.

A 29-year-old girl, G1P1, presenting with amenorrhea for 2 months, irregular vaginal bleeding for 1 month and elevated serum hCG level (83,962IU/L), was admitted to the gynecology department. The antecedent pregnancy of this patient was a full-term cesarean delivery 7 years ago. An ultrasound examination revealed a mixed echogenic mass measuring 3.2*4.5*3.9 cm in the uterine cavity, partially invading the myometrium, with honeycomb like internal echoes (**Figure 1A**), and color Doppler showed that it was filled with abundant blood flow signals (**Figure 1B**), and the spectrum of arteriovenous fistula was measured in pulsed-wave Doppler (**Figure 1C**). Meanwhile, the pelvic MRI scan depicted heterogeneous mass measuring 3.9*4.0cm in uterine cavity with hemorrhage and invasion of the myometrium (**Figure 1D**). There were no abnormal findings on gynecological examination, chest CT scan and other image examinations.

**Figure 2:**
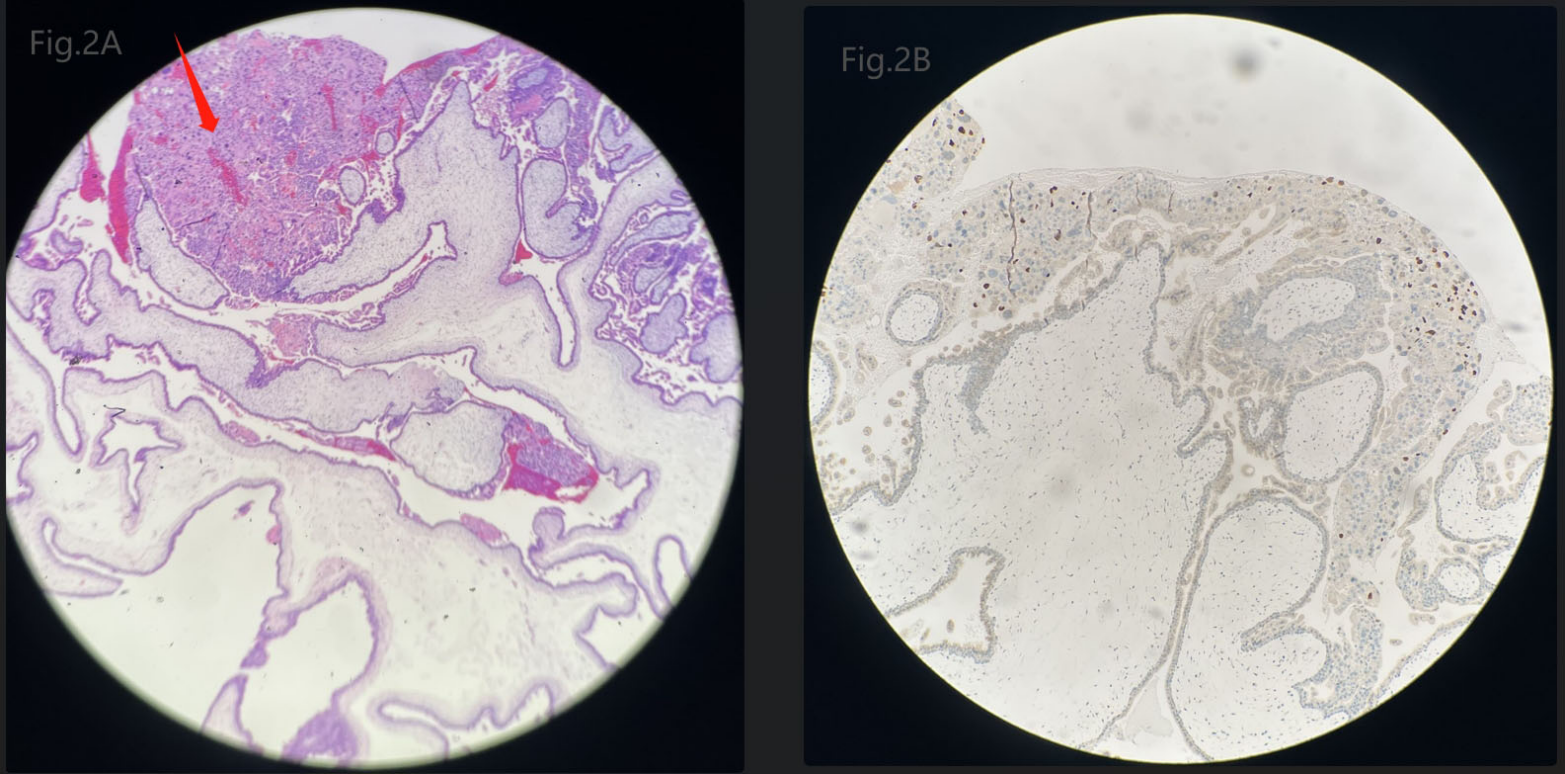
**(A)** Microscopic image of the evacuated mole tissues. Arrow points to moderate to severe trophoblast proliferation. **(B)** Immunohistochemical staining of the evacuated mole tissues identified a negative stain of p57.

**Figure 3:**
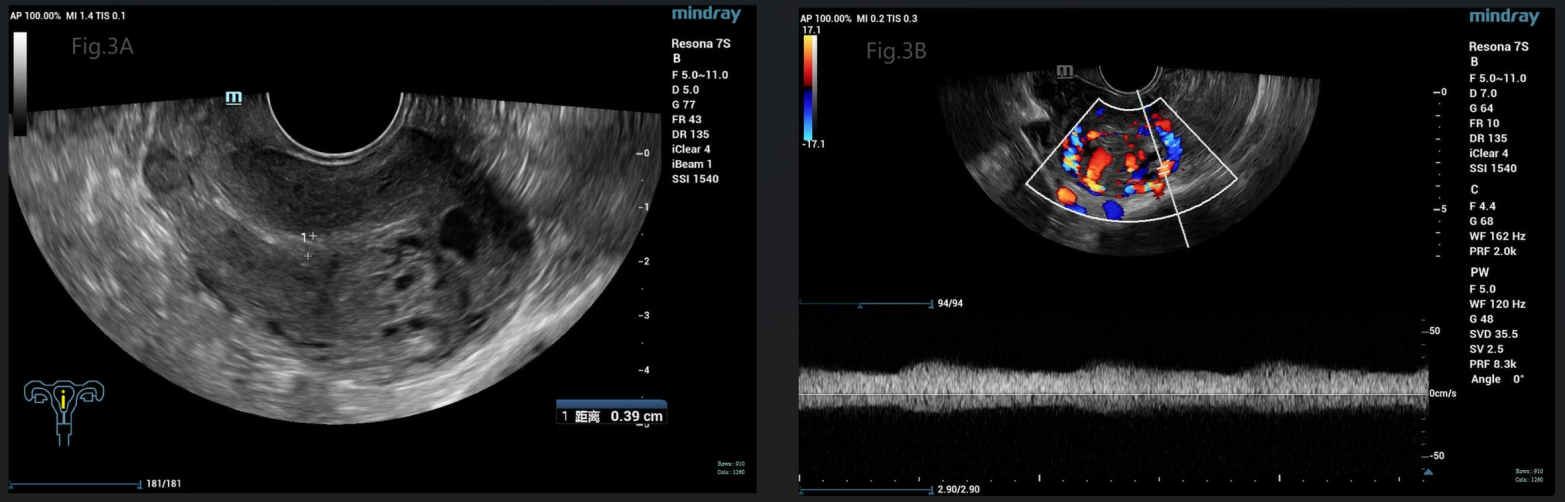
**(A)** Gray-scale pelvic sonography showed a large honeycomb like mass within the uterine fundus, close to the serosa. **(B)** Pulsed-wave Doppler detected a high-speed, low-resistance arteriovenous fistula spectrum.

The patient’s diagnosis caused controversy amongst the medical team. Someone considered it as GTN (choriocarcinoma) originating from the previous full-term pregnancy on the basis of the fact that GTN can emerge in many years or decades after a previous pregnancy. The patient, who is currently presented with abnormal vaginal bleeding, an elevated hCG level and positive images suggesting myometrial invasion, had an antecedent full-term cesarean section 7 years ago. While others disagreed with the above suspected diagnosis. They viewed it as a new onset of pregnancy which quickly progressed to a GTN, although the appearance was very rare. Because abnormal lesions occurred simultaneously in the uterine cavity and myometrium. In order to confirm the origin of the myometrial invasive mass, suction evacuation was performed after the discussion of the medical group. Histologic examination showed moderate to severe trophoblast proliferation accompanied with edematous villi (**Figure 2A**) and immunohistochemical staining identified p57 negative (**Figure 2B**), which indicated a complete molar pregnancy. The patient was reevaluated one week post evacuation. Although the hCG decreased to 26,444IU/L, ultrasound suggested honeycomb like mass filled with abundant fire-sea-like blood flow signals was seen within the uterine fundus measuring 5.3*4.8*2.7cm (**Figure 3A**). Additionally, pulsed-wave Doppler detected a high-speed, low-resistance arteriovenous fistula spectrum. (**Figure 3B**). Hence, pGTN was diagnosed as FIGO stage I with the prognostic score of 4(2 points for pre-treatment hCG concentrations, 2 points for the largest tumor mass diameter).

According to 2000 FIGO staging, a risk score of 6 and below is classified as low risk. Despite the patient’s prognostic score being low risk, EMA/CO (etoposide, methotrexate, and dactinomycin alternating with cyclophosphamide and vincristine) multi-agent chemotherapy regimen was selected in order to avoid single-agent chemotherapy resistance, taking into account the patient’s extensive uterine invasion, the high hCG level prior to chemotherapy, and the highly malignant potential of the tumor. The patient’s HCG decreased drastically to 716.3 IU/L after the first chemotherapy course, to 8.9 IU/L after the second course, and was normalized after the third course. Additional 2 courses of chemotherapy were given to minimize the risk of recurrence. The last course of additional chemotherapy was replaced by methotrexate mono-chemotherapy due to severe bone marrow suppression caused by multi-agent chemotherapy. Ultrasound after each chemotherapy course showed gradual regression of the mass, with complete disappearance of the lesion and the arteriovenous fistula at the last chemotherapy course. The woman was followed up for one year and her hCG level was normal with regular periods.

## Discussion

GTN most commonly follows a molar pregnancy but may develop after any other type of pregnancy. The varied presentations of GTN can be irregular vaginal bleeding, an enlarged and irregular uterus, bilateral ovarian enlargement, or even asymptomatic (2). pGTN (usually invasive mole, occasionally choriocarcinoma) most commonly occurs following evacuation of CHM and is usually clinically diagnosed based on a plateaued or rising hCG concentration after evacuation of mole tissues. hCG is the first effective biomarker employed in the diagnosis and follow-up of the GTN (9). During post-molar follow-up, pGTN is diagnosed by a rising serum hCG levels ≥10% for 2 consecutive weeks or a plateau in serum hCG levels for 3 consecutive weeks (3), without histologic verification. In general, pGTN diagnosis can be timely confirmed if the patient complies with the hCG monitoring protocol after expulsion of uterine molar tissues. Delayed diagnosis of GTN can lead to life-threatening complications and raise the risk of surgical intervention (10). Therefore, early diagnosis and timely implementation of chemotherapy are very important for the prognosis of GTN patients. In rare cases, pGTN can progress rapidly, presenting before the diagnosis of hydatidiform mole is confirmed, as in this case report.

Thus far, the pathogenesis of GTN still remains unknown and etiologic risk factors that contribute to the development of GTN are unclear. There are several factors that are known to influence the development of GTN from CMH including age >40 years, serum hCG>100,000 IU/mL, excessive uterine enlargement, and/or theca lutein cysts larger than 6 cm (1, 11). Prophylactic chemotherapy may reduce the incidence of pGTN for patients with above high-risk factors (1). Currently, there are increasing studies attempting to explore genetic and molecular biomarkers to predict pGTN. A cohort study demonstrated that heterozygous CHM had a higher potential for GTN than homozygous CHM (12). Braga et al. reported that the malignant transformation of CHM is closely linked to the apoptotic index, and this may be a useful biomarker to predict pGTN (13).

The wide availability of first trimester ultrasound can detect the suspicious molar pregnancy. Classic ultrasound findings of CHM include a heterogeneous uterine mass with cystic spaces (snowstorm appearance), no identifiable fetus or embryo, and no amniotic fluid. Sensitivity of pelvic ultrasound in diagnosing CMH ranges from 70% to 90%, and increase with gestational age (14–16). There is limited data on ultrasound morphologic features of gestational trophoblastic neoplasia. Pelvic ultrasound can determine the tumor burden and vascularity of the uterus and is usually conducted before treatment due to its availability and simplicity. Epstein et al. reported that the majority of uterine pGTN lesions were focal in the myometrium, with moderate to rich vascularization, which is in accordance with this case (17). Another fascinating result that was found is that tumor size larger than 4 cm was an independent predictor of methotrexate mono-chemotherapy resistance for GTN (17). This finding may support the choice of multi-agent chemotherapy in this case. The study of Lin et al. revealed that abnormal myometrial vascularization and lower uterine artery Doppler indices were correlated with GTN and lower uterine artery Doppler indices were associated with methotrexate resistance (18). Thus, ultrasound can be used both in the initial assessment in women with GTN, and to indicate the prognosis of GTN. It is worth noting that GTN lesions can only be detected by ultrasound at relatively high levels of hCG but may not be visualized at lower levels of hCG (19). In this condition, a lower uterine artery pulsatility index; presence of myometrial nodules within the myometrium or endometrium; or increased signal with power Doppler within the myometrium or endometrium may be predictive of GTN development (20). Magnetic resonance imaging, as a complementary investigation to Doppler ultrasound, is better in accessing tumor extension (21).

Although the first-line therapy for patients with low-risk GTN is single-agent chemotherapy, there are many studies that support further stratified management of low-risk GTN due to the challenge of single-agent chemotherapy resistance. A UK study showed that single-agent chemotherapy cure rate in low-risk GTN was significantly negatively correlated with the risk score, with a 45% cure rate in patients with a score of 4 (22). A US study reported that GTN patients with prognostic scores of 2-4 had a 2.02(P=0.027)times higher risk of single-agent chemotherapy resistance than those with scores of 0-2, and the risk of resistance with pretreatment hCG ≥10,000 IU/L was 2.62(P=0.002)times higher than those with less than 10,000IU/L (23). A study in China revealed that high risk factors for single-agent chemotherapy resistance in patients with low-risk GTN included a pre-chemotherapy hCG ≥ 4000IU/L, the presence of invasive lesions in the uterine corpus, and a FIGO prognostic score ≥ 5 (24). In this case, pre-chemotherapy hCG was 26,444 IU/L, the mass was extensive in the uterine corpus, and the malignancy progressed rapidly, taking these factors in to consideration, a multi-agent chemotherapy regimen was implemented and the efficacy was satisfactory.

Uterine arteriovenous malformation (AVM) following GTN is a rare condition. AVM can trigger chronic vaginal bleeding or life-threatening heavy bleeding, which can occur even after the complete regression of GTN after chemotherapy (25). The formation of uterine AVM in GTN is associated with a disorganized trophoblastic proliferation, increased angiogenesis caused by high levels of hCG, finally uterine curettage (26). The proliferation of trophoblastic tissue may destroy blood vessel walls and connect arteries and veins, thereby facilitating the formation of uterine AVM (27). In this case, the uterine AVM was detected by ultrasound at the patient’s initial visit, with mild abnormal vaginal bleeding and disappeared at the fifth chemotherapy course. Because of the rarity of uterine AVM and GTN coexisting, detailed information and related ultrasound images are included in this case presentation. More studies are expected to elaborate the role of uterine AVM in the diagnosis and management of GTN.

In summary, this case reported an unusual occurrence presenting with concurrence of CMH and pGTN. Despite being diagnosed with low-risk GTN, the patient received a combination chemotherapy regimen with EMA/CO due to the stratified management of low-risk patients. At the same time, this patient had a concomitant AVM, which is a very rare condition. Enlightenments from this case are revealed as following: 1. Although pGTN usually diagnosed weeks to months post evacuation of molar tissues by hCG monitoring, in rare cases, rapid progression from molar pregnancy to pGTN can occur just as this case. This case suggests that the usual hCG monitoring protocol for diagnosis of pGTN is unfavorable to the early diagnosis and management in this specific condition. Imaging, especially sonography, in combination with hCG monitoring, plays a key role in the diagnosis and treatment of this concern. 2. Low-risk GTN should be treated individualized. Multi-agent chemotherapy may be more favorable in low-risk GTN with a large tumor size, high hCG level, low uterine artery Doppler indices and high FIGO prognostic score. But further studies are expected to explore the concrete parameter. 3. AVM can occur simultaneously with GTN, which may indicate an underlying association between the two disorders.

## Data availability statement

The original contributions presented in the study are included in the article/supplementary material. Further inquiries can be directed to the corresponding author.

## Ethics statement

The studies involving humans were approved by the ethics committee of Hangzhou First people’s Hospital. The studies were conducted in accordance with the local legislation and institutional requirements. The participants provided their written informed consent to participate in this study. Written informed consent was obtained from the individual(s) for the publication of any potentially identifiable images or data included in this article.

## Author contributions

JQ: Resources, Writing – original draft. KG: Writing – review & editing. LS: Methodology, Writing – review & editing.
